# AI can empower agriculture for global food security: challenges and prospects in developing nations

**DOI:** 10.3389/frai.2024.1328530

**Published:** 2024-04-25

**Authors:** Ali Ahmad, Anderson X. W. Liew, Francesca Venturini, Athanasios Kalogeras, Alessandro Candiani, Giacomo Di Benedetto, Segun Ajibola, Pedro Cartujo, Pablo Romero, Aspasia Lykoudi, Michelangelo Mastrorocco De Grandis, Christos Xouris, Riccardo Lo Bianco, Irawan Doddy, Isa Elegbede, Giuseppe Falvo D'Urso Labate, Luis F. García del Moral, Vanessa Martos

**Affiliations:** ^1^Research Institute for Integrated Coastal Zone Management, Polytechnic University of Valencia, Grau de Gandia, Valencia, Spain; ^2^International Space University, Strasbourg, France; ^3^Institute of Applied Mathematics and Physics, Zurich University of Applied Sciences, Winterthur, Switzerland; ^4^TOELT LLC, Dübendorf, Switzerland; ^5^Industrial Systems Institute, ATHENA Research Center, Pátrai, Greece; ^6^DNAPHONE Srl, Parma, Italy; ^7^EnginLife-Engineering Solutions – EnginLife, Rome, Italy; ^8^Afridat UG, Bonn, Germany; ^9^NOVA IMS, Universidade Nova de Lisboa, Campus de Campolide, Lisbon, Portugal; ^10^Department of Electronic and Computer Technology, University of Granada, Granada, Spain; ^11^GRANIOT Satellite Technologies S.L, Granada, Spain; ^12^Emmanouil Orfanos Kai Sia EE (ORF), Patra, Greece; ^13^Fgtech Srls, Bologna, Italy; ^14^Gaia Robotics Idiotiki Kefalaiouxiki Etaireia, Patras, Greece; ^15^Department of Agricultural, Food and Forest Sciences, University of Palermo, Viale delle Scienze, Palermo, Italy; ^16^Department of Mechanical Engineering, Universitas Muhammadiyah Pontianak – Universitas, Kalimantan Barat, Indonesia; ^17^Saeio Global Ltd, Agege Lagos, Nigeria; ^18^EKROME Srl, Rome, Italy; ^19^Department of Plant Physiology, Institute of Biotechnology, University of Granada, Granada, Spain

**Keywords:** edge intelligence, agribusiness, Agriculture 5.0, sustainability, food security

## Abstract

Food and nutrition are a steadfast essential to all living organisms. With specific reference to humans, the sufficient and efficient supply of food is a challenge as the world population continues to grow. Artificial Intelligence (AI) could be identified as a plausible technology in this 5th industrial revolution in bringing us closer to achieving zero hunger by 2030—Goal 2 of the United Nations Sustainable Development Goals (UNSDG). This goal cannot be achieved unless the digital divide among developed and underdeveloped countries is addressed. Nevertheless, developing and underdeveloped regions fall behind in economic resources; however, they harbor untapped potential to effectively address the impending demands posed by the soaring world population. Therefore, this study explores the in-depth potential of AI in the agriculture sector for developing and under-developed countries. Similarly, it aims to emphasize the proven efficiency and spin-off applications of AI in the advancement of agriculture. Currently, AI is being utilized in various spheres of agriculture, including but not limited to crop surveillance, irrigation management, disease identification, fertilization practices, task automation, image manipulation, data processing, yield forecasting, supply chain optimization, implementation of decision support system (DSS), weed control, and the enhancement of resource utilization. Whereas AI supports food safety and security by ensuring higher crop yields that are acquired by harnessing the potential of multi-temporal remote sensing (RS) techniques to accurately discern diverse crop phenotypes, monitor land cover dynamics, assess variations in soil organic matter, predict soil moisture levels, conduct plant biomass modeling, and enable comprehensive crop monitoring. The present study identifies various challenges, including financial, infrastructure, experts, data availability, customization, regulatory framework, cultural norms and attitudes, access to market, and interdisciplinary collaboration, in the adoption of AI for developing nations with their subsequent remedies. The identification of challenges and opportunities in the implementation of AI could ignite further research and actions in these regions; thereby supporting sustainable development.

## 1 Introduction

At present, the practical applications of Artificial Intelligence (AI) in the field of agriculture are somewhat limited to the developed countries (Goralski and Tan, 2020). While the swift progress within the AI domain might instill concerns about job displacement in more developed nations, it holds the potential to be viewed as a promising opportunity for developing countries (Lohr, 2018). However, the importance of AI in the agriculture sector cannot be overlooked given its potential in augmenting crop production, disease monitoring, fertilizer and irrigation management, optimized harvesting, and post-harvest managerial efficiencies (Dharmaraj and Vijayanand, 2018; Eli-Chukwu, 2019; Lioutas et al., 2021; Shen et al., 2022). While the presence of the digital divide within the agriculture sector has been documented in previous reports along with its prospective solutions (Rotz et al., 2019; Lioutas et al., 2021), there is still a significant scope for enhancement. Therefore, there is a great need to adopt a holistic approach to overcome the digital divide for an equal implementation of AI in developed and developing countries so as to support the United Nations Sustainable Development Goals (UNSDG) of zero hunger by ensuring food safety and security.

**Figure 1:**
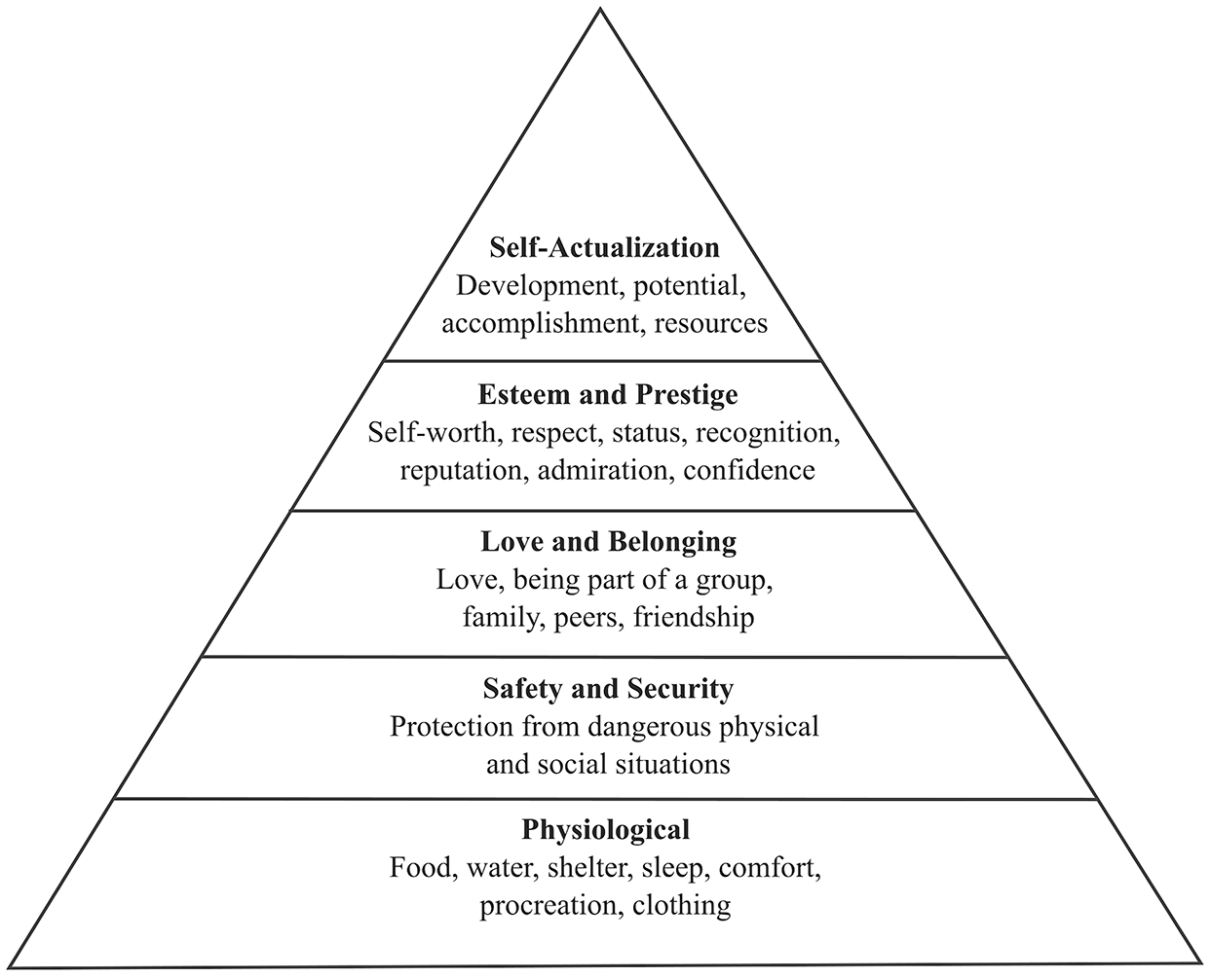
Abraham Maslow’s hierarchy of needs.

American psychologist Maslow (1974) first postulated “Maslow’s Hierarchy of Needs” outlining the theory of psychological health which included food as its essential element (Maslow, 1974; Numonjonovich, 2022). This was categorized as a physiological need. Maslow’s pyramid (Figure 1) illustrates this need located at its base. This theory remains pertinent to a great extent as it highlights the essential element of survival for all living beings. Fast forward to 2020, a pandemic era affecting the global population where the vast inequality between the have and have-nots was experienced by many, the wealthy and the needy, and those barely surviving due to the unequal distribution of wealth; situation stemming from disparities in opportunities and development potential (Bayati et al., 2022). The provision of food and nutrition in the developing world could be approximated as an analogous scenario to the inequal distribution of COVID-19 vaccines (Tatar et al., 2021; Bayati et al., 2022).

Most advanced economies have a well-established ministry overlooking the development of scientific innovation and engineering, this includes the advancements in the fields of AI and Machine Learning (ML). These technologies were pioneered in the areas of military, defense and space exploration with their spin-offs applied to the primary sector such as agriculture (Arakpogun et al., 2021). However, one of the main objectives of these technologies is to ensure higher crop production to meet the internal and external demands. These technologies have the potential to improve livelihoods in the developing world. An example would be the identification of suitable farmlands to meet and harvest the agricultural demands of these populations. AI applications in agriculture increase the efficiency of crop production through the identification of ideal farmlands and terrains, efficient water management, early disease detection, and optimal use of input resources, thus eliminating the lag-time that otherwise would have encountered a trial-and-error approach (Dharmaraj and Vijayanand, 2018; Eli-Chukwu, 2019; Sharma et al., 2023).

The economic gains due to the implementation of AI could further expand its potential. For instance, it has been predicted that AI would result in 10.3% higher Gross Domestic Product (GDP) for United Kingdom, thus accounting for an extra 232 billion pounds (approximately $317 billion USD) (PWC, 2022). Similarly, according to World Economic Forum (WEF), investment by worldwide corporations in AI surged by 40% from 2019 to 2020, reaching a total of $67.9 billion (Forum, 2022). Whereas AI investments are expected to reach €22.4 billion (approximately $26.5 billion) in the European Union by 2025 (Alliance, 2020). On the contrary, developing countries struggle to secure funding. AI related initiatives are either minimal or completely absent in developing nations (Pro, 2018; Loucks et al., 2019). For instance, in African countries the process of securing loans for digitalization is quite cumbersome leading to its abandonment by the farmer or small agriculture firms (Aguera et al., 2020). It has been reported that a total of $140 million of seed-funding across the whole of Africa for AI startups was secured (Hamdan et al., 2021). The significant differences indicate that developing countries are behind by a substantial percentage in terms of funding and gains for AI initiatives compared to more developed regions.

The focus of the current study centers on fundamental inquiries concerning the identification of adoption barriers to AI technologies in developing countries along with their subsequent mitigation and tailoring strategies. This would ultimately strengthen sustainable food production. Whereas the objective of this review is to explore the in-depth potential of AI in the agriculture sector. Similarly, it aims to emphasize the proven efficiency and spin-off applications of AI in the advancement of agriculture for the developing and under-developed countries (For reader convenience, we will subsequently refer to developing and under-developed countries as “developing countries”). The identification of challenges and opportunities in the implementation of AI could ignite further research and actions in these regions. Furthermore, this review also details the available technologies, and identifies plausible methods that could enable efficient agriculture production and management as we progress in achieving food and nutrition equality in the 21st century.

This study is organized into ten distinct sections. The second section details the methodology, the third addresses the necessity of AI technologies in agriculture. The fourth section systematically examines plausible AI applications, drawing insights from previous research. Subsequently, the fifth section scrutinizes the paradigm of edge intelligence, specifically within the agricultural context, followed by an analytical discourse on the economic and tangible merits of AI. Following that, the study discusses solutions for enhancing food safety and security. The eighth section outlines challenges in AI adoption along with their potential solutions. The ninth section underscores an integrative approach, focusing on Agriculture 5.0. Finally, a concise summary of the study is provided.

## 2 Methodology

This study was driven by the imperative to address four pivotal inquiries at the intersection of AI, agriculture, and sustainability. To systematically investigate these questions, an extensive examination of scholarly materials was conducted, utilizing reputable databases such as Web of Science (WOS), Scopus, and Google Scholar. The formulated research questions are as follows:

- What are the prevailing imperatives propelling the incorporation of AI technologies in agricultural practices?
- What insights can be gleaned from previous studies regarding potential AI applications in agriculture?
- In what ways can AI contribute to advancing food safety and security in the agricultural sector?
- What challenges influence the adoption of AI for agricultural operations, particularly in developing countries?

**Figure 2:**
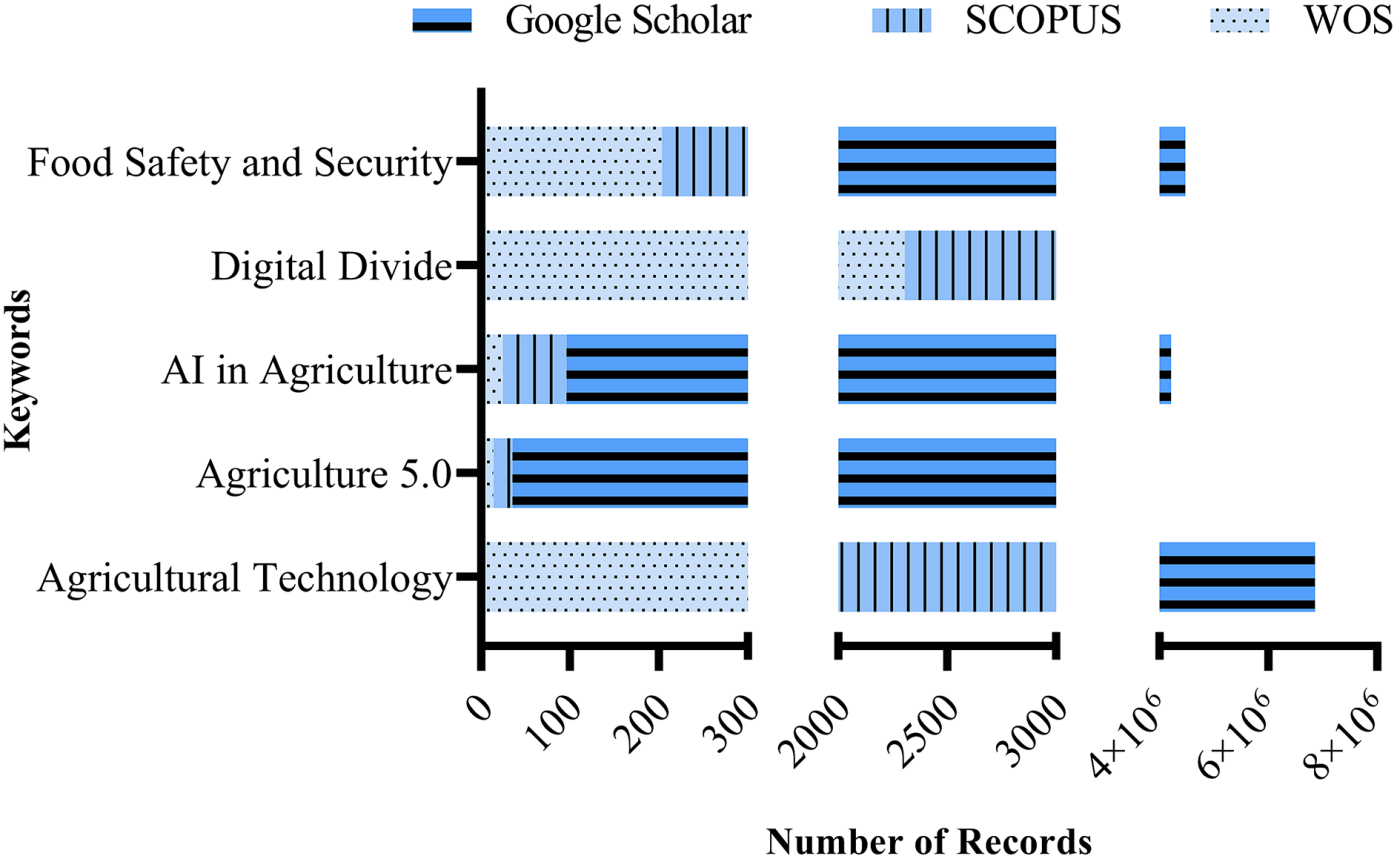
Summary of records for each keyword found on Scopus, Web of Science (WOS), and Google Scholar databases.

To conduct the research, specific keywords including “Agricultural Technology,” “Agriculture 5.0,” “AI in Agriculture,” “Digital Divide,” and “Food Safety and Security” were employed. The initial search results revealed a significant abundance of records for the keyword “Digital Divide,” followed by “Agricultural Technology” on Scopus and WOS databases (Figure 2). Conversely, “Agriculture 5.0” yielded minimal documents in both databases. Google Scholar, while presenting millions of documents for each keyword, was excluded from further analysis due to its lower precision and generality.

**Figure 3:**
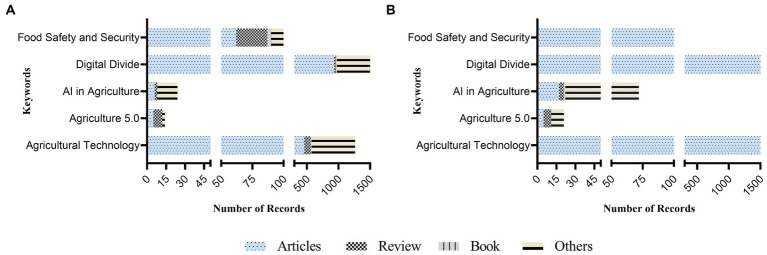
Summary of research articles, review papers, and books for corresponding keywords, where **(A)** represents data retrieved from Web of Science (WOS) and **(B)** represents data retrieved from SCOPUS.

**Figure 4:**
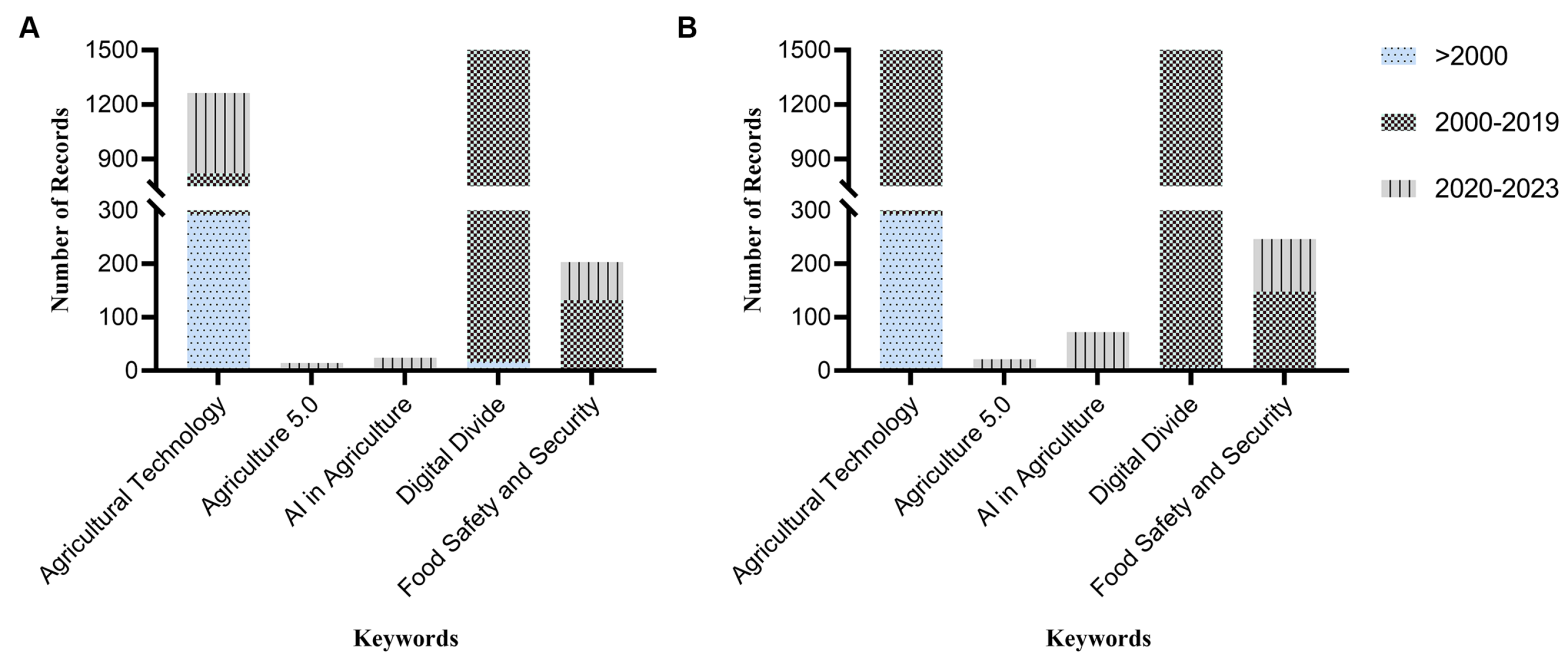
Summary of research articles, review papers, and books pertaining to specific keywords across different time periods, where **(A)** represents data retrieved from Web of Science (WOS) and **(B)** represents data retrieved from SCOPUS.

In terms of document types, Scopus exhibited a higher number of research articles, review papers, and books compared to WOS. For example, the keyword “AI in Agriculture” yielded 17 research articles in Scopus versus 6 in WOS. The document types were categorized into “Others” for materials not falling into the research article, review paper, or book categories (Figure 3). An exploration of publication volumes across three distinct periods—before 2000, between 2000 and 2019, and from 2020 to 2023—highlighted a notable surge in interest and research output in AI, food safety and security, agriculture 5.0, and agricultural technology (Figure 4). This trend indicates an escalating focus on these keywords over the last decade.

Following the comprehensive collection of pertinent studies, a rigorous filtration process was employed to ensure the relevance and depth of the investigation. Research articles, review papers, and books that directly addressed the posed research questions in the agricultural domain were considered. Given the innovative nature of concepts such as “Agriculture 5.0” and “AI in Agriculture,” particular emphasis was placed on studies conducted over the last five years, with additional attention given to those published within the past decade. Additionally, valuable insights from reputable sources, such as the World Health Organization (WHO) and Food and Agriculture Organization (FAO) websites, were incorporated. The citation of specific studies was conducted impartially, aiming to support the narrative on the utilization of AI in agriculture for enhancing food safety and security, particularly in developing nations, without implying endorsement or validation of specific information.

## 3 The need for AI

**Figure 5:**
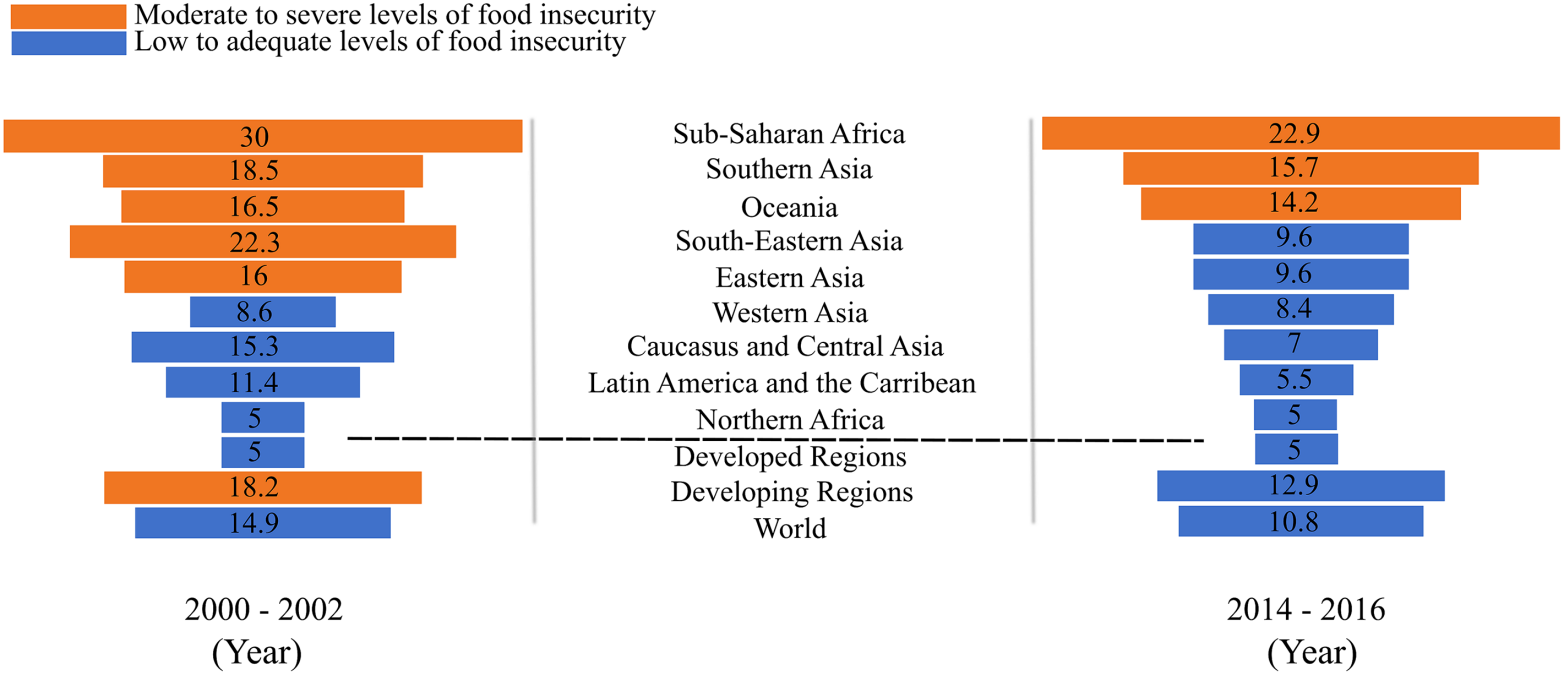
The proportion of undernourished people worldwide between the study periods of 2000–2002 and 2014–2016 (percentages). Data retrieved from the United Nations Sustainable Development Goals Report (2016).

**Figure 6:**
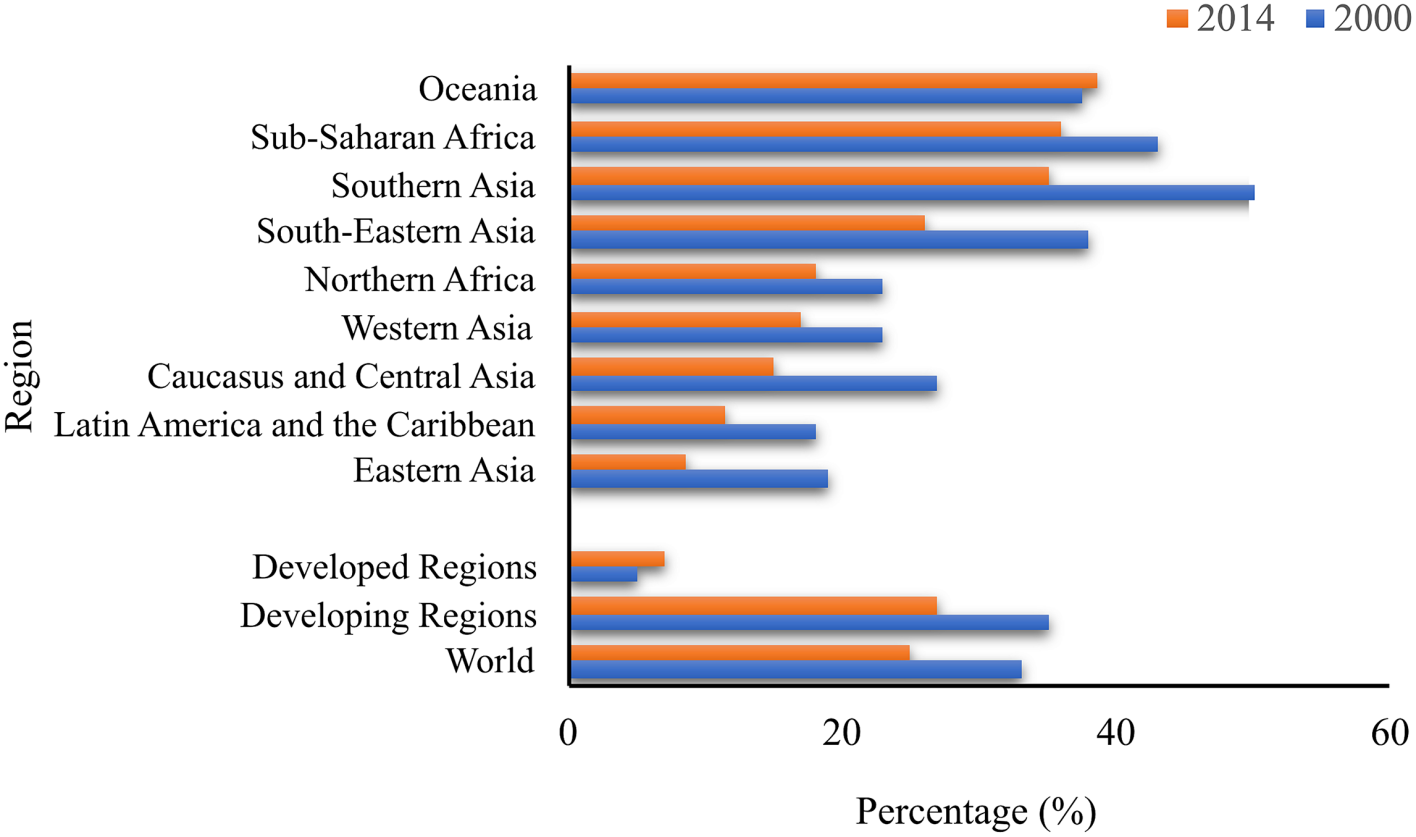
The proportion of children under age five with stunted growth, 2000 and 2014 (percentages). Data retrieved from the United Nations Sustainable Development Goals Report (2016).

AI in agriculture is a crucial factor in bridging the hunger gap in the developing world. The developing region recorded 780 million (12.9%) of undernourished people as compared to 795 million (10.8%) worldwide in a longitudinal study over the period of 2014–2016 (United Nations Sustainable Development Goals Report, 2016). These figures illustrate that over 98% of the undernourished population is from developing regions (Figure 5). This is corroborated with the number of malnourished children in these regions as demonstrated in Figure 6. This data indicates the lack of resources to meet the basic needs of the population. These disparities are owing to various factors, chief among them are remote locations, level of economic development and poor infrastructure.

The preemptive measures to the existential threats manifested as climate change and environmental regression have resulted in a pivotal initiative that envisions a modern, resource-efficient, and competitive economy ensuring the achievement of the triad of core tenets of the European Green Deal: no net emissions of greenhouse gasses by 2050, economic growth decoupled from resource utilization, and that no individual or location is left out (Commission, E, 2021a). Reports from various agencies including the FAO emphasize the greater need of enhancing food production to meet the augmenting grain consumption needs considering the global population growth (Singh and Kaur, 2022). This challenge might not be addressed by merely increasing the production land or snowballing the breeding programs. Rather, a holistic approach for an integrated management of agriculture production is needed. Likewise, AI’s potential in agriculture extends to predictive analytics, genomics, phenomics, plant breeding, virtual assistance, DSS, and the integration with blockchain technology. Predictive analytics aids in risk management, productivity enhancement, and precision farming. In genomics and phenomics, AI contributes to transformative advancements, facilitating tasks such as tissue-specific gene expression prediction and cultivar selection. Virtual assistance through AI-powered chat bots and the use of DSS for data-driven decision-making are rising trends. The integration of AI with blockchain technology enhances traceability and data management, addressing challenges in supply chain management. Moreover, AI plays a supplementary role in various domains, including weed identification, forestry and livestock management, farm robotics, agricultural remote sensing, mechanical pollination, and crop insurance, contributing to increased agricultural production and meeting the demands of vulnerable populations (Singh and Singh, 2020; Ahmad et al., 2022b). In this regard, AI holds a vast potential.

The deployment of AI-enabled agriculture could greatly aid in narrowing the gap and meet the essential nutritional needs. These can be viewed through a two-pronged approach. One is collaboration between wealthier nations using AI to improve their own agriculture or using AI extensively in developed nations to boost productivity and export surplus to developing regions. The second approach involves a combination of both strategies. Through these advanced technologies, it is projected that agricultural productivity can be augmented by 70 percent by 2050 (Research, B, 2015; Martos et al., 2021).

Undeniably, Agriculture 5.0 is present in most developing and developed nations such as China, Argentina and Israel through various stages of implementation, and its use is projected to increase in the near future through the multitude of methods in cultivating food supplies (Review, 2020). The application of Agriculture 5.0 ranges from small scale indoor cultivation to large-scale commercial farmland. The ingenuity of these technologies allows for the monitoring of vital statistics of essential crops. For example, VineScout is a mobile robot that monitors moisture levels of soil and detects diseases in vineyards (Saiz-Rubio and Rovira-Más, 2020). These were achieved through a variety of devices including handheld devices and mobile robots (Saiz-Rubio and Rovira-Más, 2020; AgriFarming, 2022). The application of AI in agriculture offers numerous benefits, particularly in the development of efficient and intelligent irrigation systems. The use of Artificial Neural Network (ANN) models, such as those employed by Hinnell et al. (2010) and Arif et al. (2013) for soil moisture detection and intelligent irrigation, showcases the potential to ensure precise and optimal watering of crops. Additionally, the integration of Internet of Things (IoT) and ML, as demonstrated in the smart irrigation system by Tace et al. (2022), further enhances agricultural practices. These advancements not only facilitate timely irrigation but also contribute to water conservation, paving the way for sustainable agriculture.

Taking into consideration the broader context of malnutrition and hunger, developing countries such as in Sub-Saharan African region, emerge as regions endemic with chronic hunger. The World Food Program’s longitudinal study between the years of 2018 and 2020 is strongly corroborated by the trend published by the UN study between the years of 2014 and 2016 (United Nations Sustainable Development Goals Report, 2016; Programme, 2021). However, the COVID-19 pandemic resulted in a spike of the population prevalent to hunger and undernourishment with 1 in 10 (811 million) of the global population experiencing a short supply of food (Programme, 2021). This requires the urgency of remedial action in this region. These arduous circumstances provide an opportunity for the application of AI in agriculture.

Climate change is posing severe challenges of water scarcity across the globe. AI could efficiently be applied to mitigate such abiotic stresses. The use of AI could help devise efficient and smart irrigation systems along with precise and optimum irrigation of crops. For instance, implementation of ANN based models could be used for crop-irrigation monitoring, thereby contributing toward efficient water use (Arif et al., 2013). Furthermore, AI supports digital farming that aims to limit carbon footprint and contribute toward environmental sustainability. Similarly, incorporation of AI into agriculture sector could gain higher economic gains (Ryan et al., 2023). It has been reported that a higher tendency of firms adopting AI was witnessed during the last decade (Davenport et al., 2020). Consequently, envisaging the agriculture sector with AI technologies would create novel economic opportunities locally and globally. Additionally, Agricultural AI concentrating on external elements employs data related to crop values, market patterns, consumer preferences, needs, and visual aspects (Dharmaraj and Vijayanand, 2018). This has the potential to enable farmers to take market-savvy actions more effectively.

## 4 Applications of AI in agriculture

AI could play a crucial role in modern day agriculture. The developed nations are leading the way of AI applications in agricultural domain, which could be used as an example by developing regions. Currently, AI is being applied for crop monitoring, irrigation, disease detection, fertilization, task execution, automation, image processing, data processing, yield simulations, DSS, weeds management, and optimizing input resources among others (Table 1). However, it is imperative to acknowledge the constrained efficacy of AI within these contexts. This disparity could be addressed by increasing research and experimentation, particularly at national and international level.

**Table 1:** Summary of Artificial Intelligence (AI) applications in agriculture along with corresponding methodology, findings, and challenges.

Applications of AI in Agriculture	Key Findings	Methodology	Key Outcomes	Limitations and Challenges	References
Crop monitoring and irrigation	AI enhances irrigation systems. Smart irrigation systems use ANN and IoT.	ANN based models for soil moisture. Use of IoT for smart irrigation.	Efficient irrigation and water savings. Smart irrigation through IoT and ML.	Need for extensive datasets and computational resources.	Arif et al. (2013), Sinwar et al. (2020), Tace et al. (2022)
Disease detection	AI aids timely identification of diseases. High accuracy in disease detection through AI.	Image based diagnostics using AI. Agriculture expert systems using ANN and computer vision.	Timely disease identification. High accuracy in disease detection.	Challenges include dataset requirements and computational resources.	Balleda et al. (2014), Ferentinos (2018), Barbedo (2019), Fang et al. (2020), Singh et al. (2020)
Predictive analytics	Supports risk management and precision farming. Application in crop yield estimation.	Integration of predictive analytics in agriculture.	Improved risk management and precision farming. Challenges in data complexity and adoption.	Need for substantial data and limited farmer adoption.	Vijayabaskar et al. (2017), Kolipaka (2020), Yoosefzadeh-Najafabadi et al. (2021), Kumar et al. (2022)
Genomics and plant breeding	AI enhances gene expression and cultivar selection. ML models for gene expression and cultivar prediction.	ML models for gene expression and pattern identification. CNN for crop trait classification.	Improved gene expression and cultivar selection. CNN for crop trait classification.	Challenges include complex algorithms and data resistance from breeders.	Pound et al. (2017)Koirala et al. (2019), Washburn et al. (2019), Cho et al. (2022), Jafari et al. (2022), Pallante et al. (2022)
Chat bots and virtual assistance	AI powered chat bots for disease identification. Virtual assistance for agronomic inquiries.	Natural language conversation algorithms.	Speeds up disease identification and provides personalized conversation.	Prone to errors, requiring continuous ML model training.	Conesa-Muñoz et al. (2016), Talaviya et al. (2020), Singh and Kaur (2022)
Decision support systems (DSS)	AI based DSS for optimized task execution. DSS for herbicide treatment and farm operations.	Implementation of DSS for task allocation. Integrated DSS for disease identification.	Optimal task allocation and precise disease identification.	Challenges include farmer knowledge gaps and DSS limitations.	Navarro-Hellín et al. (2016), Alsalam et al. (2017), Kukar et al. (2019), Li et al. (2020), Zhai et al. (2020)
Blockchain for traceability	Blockchain ensures traceability of agricultural produce. Data integrity and transparency in the supply chain.	Integration of blockchain with AI for traceability.	Ensures traceability and data integrity in the supply chain.	Limited integration of blockchain with AI and IoT in agriculture.	Kshetri (2019), Singh and Singh (2020), Jabbar et al. (2021), Bermejo et al. (2022)
Edge intelligence	Combining AI and edge computing for crop monitoring. Applications in real time weed mapping.	Use of edge computing for crop monitoring. Detection of pine wilt disease using edge computing.	Shows promise in crop monitoring and disease detection. Challenges in processing power and scalability.	Constraints in processing power, scalability, and integration complexities.	Shekhar et al. (2017), Deng et al. (2020), Kawai and Mineno (2020), Li et al. (2021), Liu et al. (2021), Rani et al. (2023)

AI’s diverse applications in agriculture could contribute to the development of efficient and intelligent irrigation systems, ensuring precise and optimal watering of crops. For instance, ANN based model was used to detect the soil moisture level with subsequent alert for irrigation (Arif et al., 2013). Similarly, a novel approach, named Neuro-Drip, to visualize the spatial and temporal subsurface water, using ANN and statistical analysis was reported by Hinnell et al. (2010). Likewise, a support system for intelligent irrigation to estimate the irrigation need for one week using AI was also reported (Sinwar et al., 2020). This could not only facilitate the timely irrigation but also facilitate the means to save water. Recently, a highly sophisticated smart irrigation system was developed using IoT and ML (Tace et al., 2022). Authors collected data through humidity, temperature, and rain sensors and after proper analysis presented it in the form of a web application. Apart from these, there are numerous studies involving AI aimed at smart irrigation to save water and improve the efficiency of irrigation systems; thereby paving the way to sustainable agriculture.

Plant diseases and pests are a big constraint to higher crop yields and sustainable crop production. Every year the agriculture sector encounters billions of dollars loss due to plant diseases (Ahmad et al., 2023). One of the possible ways to address this problem is the timely identification of crop diseases. The diagnostic feature of AI, through images of a leaves, has been shown to provide an accuracy of 75% as opposed to alternative diagnostic methods (Barbedo, 2019; Martos et al., 2021). AI has been shown to be efficient in this task of disease identification. For example, an agriculture expert system “Agpest” was developed for wheat and rice crop pest management by employing ANN, genetic algorithm (GA) and computer vision system (Balleda et al., 2014). An accuracy of 99.53% in disease detection using a convolutional neural network (CNN) for 25 different plants was reported by (Ferentinos, 2018). Likewise, a remarkable system for non-destructive and visual disease detection was developed for 13 plant species and was named as “PlantDoc” (Singh et al., 2020). Equally, the use of ResNet-50 with an accuracy of 95.61% in plant disease and pest detection has also been reported (Fang et al., 2020). Numerous techniques exist based on AI techniques for plant disease and pest identification (Pandey et al., 2022; Zheng et al., 2022; Singh et al., 2023) (Nagasubramanian et al., 2019; Thenmozhi and Reddy, 2019; Liu and Wang, 2021; Xu et al., 2022).

Nonetheless, the utilization of AI techniques for plant disease detection necessitates extensive datasets encompassing both diseased and healthy plants. Despite the existence of various datasets like the PlantVillage dataset (PVD), there remains a requirement to expand the creation of more accessible datasets with diverse parameters. Similarly, notable limitations in deploying AI techniques for disease detection include the demand for increased computational resources, substantial financial investment, ongoing maintenance expenditures, and the availability of adequately trained personnel.

Predictive analytics is another promising domain of AI for agriculture. Predictive analytics support planning for risk management, gaining apex productivity, improving farmers’ performance, fulfilling customer demands, and improving crop management (Vijayabaskar et al., 2017; Gupta and Malik, 2022). In addition to this, predictive analytics finds its application in precision farming, soil nutrients level estimation, predictions of crop yields, inventory management, and the facilitation of DSS (Kolipaka, 2020; Chandraprabha and Dhanaraj, 2021; Kumar et al., 2022). Mostly agricultural firms from developed countries are exploring this domain of AI for increased gains and better supply chain management. However, its direct use by the farmers is still in its nascent phase. Huge amounts of data, complicated data processing, and lack of training are a few of the prominent challenges of adopting predictive analytics in agriculture, particularly for developing nations. In addition, lack of historical data and heterogeneity of agricultural data impede the use of predictive analytics in agriculture.

Cultivars characterized by increased yield potential, enhanced quality attributes, and resilience to adverse climatic conditions are desired globally. In pursuit of these objectives, high-throughput genomics, plant breeding methodologies, and high-throughput phenotyping techniques emerge as relevant technological avenues. The application of AI has brought about transformative advancements across each of these domains. For example, tissue-specific gene expression, in maize, based on protein sequences and deoxyribose nucleic acid (DNA) promoter was achieved through ML models with an accuracy of up to 95% (Cho et al., 2022). Similarly, ML models of “ortholog contrasts” and “gene-family guided splitting” have also been reported for predicting the messenger ribonucleic acid (mRNA) expression (Washburn et al., 2019). Furthermore, ML approaches facilitate the pattern identification in the selection of cultivars with desired characteristics. For example, genetic algorithm (GA) along with a hybrid generalized regression neural network (GRNN) was successfully devised for predicting and optimizing the complex, difficult, and non-linear *in vitro* adventitious rooting of Bluecrown Passionflower (Jafari et al., 2022). Another example is the use of CNN to classify and detect wheat spikes and spikelets for crop development with up to 95.91 and 99.66% accuracy, respectively, (Pound et al., 2017). Crop production traits have also been investigated using deep learning methodologies for the detection of fruits and consequently for estimating yield (Koirala et al., 2019). Additionally, deep neural network (DNN) and ensemble-bagging algorithms were used to predict yield and biomass of soybean (Yoosefzadeh-Najafabadi et al., 2021). As well, ML algorithms are developed and applied for detecting food taste; thus serving as an electronic tongue (Pallante et al., 2022). In short, AI has found its application from genomics to phenomics for devising appropriate data analytics (van Dijk et al., 2021), which could be further exploited for ensuring higher crop yields and sustainable agriculture.

In spite of the manifold advantages, AI encounters several limitations in its application to genomics, phenomics, and plant breeding. An illustrative instance is the intricate nature of ML algorithms. Geneticists and breeders might not necessarily be experts in data analysis techniques for devising relevant breeding strategies. Lack of historical data and the necessity of huge sets of data for model training could be another limitation. Moreover, many breeders could be reluctant to use AI for a trait of interest due to socio-economic or personal preferences. Also, AI algorithms could not be suitable for all or generalized for all scenarios.

The use of virtual assistance or chat bots to answer the simple questions of farmers is also on the rise. The AI powered chat bots use natural language conversation algorithms to provide more personalized conversation. The use of chat bots includes but not limited to disease identification via image analysis, retail, media, and general inquiries related to agronomic practices (Talaviya et al., 2020; Momaya et al., 2021; Ong et al., 2021; Chandolikar et al., 2022). This could speed up the management process on farmers’ end. For instance, identification of an infection could help farmers with the timely use of perspective insecticide. However, the chat bots are prone to errors and sometimes could produce inaccurate results of queries. Therefore, further training of ML models is needed.

Traditionally, agricultural experts are sought after for making important decisions for agricultural management. However, due to time, expertise, and personnel availability constraints data-based decisions using AI are on the rise. In this regard, DSS is an AI based platform that assists in precise and evidence-based decisions for agricultural operations. For example, the implementation of DSS to optimize task execution through ground and aerial vehicles, aimed at enhancing crop production has been reported previously (Conesa-Muñoz et al., 2016). The system was found efficient in generating optimal task allocation for site-specific herbicide treatment missions. Likewise, an integrated DSS was developed to accurately identify the precise location of disease occurrence in a crop field with the subsequent action of herbicide spray (Alsalam et al., 2017). In addition, several DSS have been devised for providing guidance for farm operations, herbicide treatment, fertilizer and irrigation management, yield estimations, designing agricultural machinery travel paths, and planning routes for drones (Navarro-Hellín et al., 2016; Kukar et al., 2019; Li et al., 2020; Zhai et al., 2020; Saqib, 2021). Nonetheless, farmers lack of knowledge about DSS, variable input needs of farmers, limited connectivity, interoperability, and performance limitation of DSS to specific task are some critical challenges for employing DSS.

With the advancement in AI, the application of QR codes can significantly support the sustainability of agricultural produce through the use of blockchain technology. For example, elemental data on the concentration of carbon dioxide (CO_2_) can be identified from the QR codes imprinted on food labels, enabling the best before date to be identified (Bermejo et al., 2022). Blockchain enables the traceability of agricultural produce, from farm to fork, in a literal sense. The aggregate elements of the agricultural produce such as the location, weather, date of harvest, transport, and best by dates are cryptographically secured and well documented through this form of information storage (Jabbar et al., 2021; Bermejo et al., 2022; Noor et al., 2022). Such applications enable data integrity and traceability of the supply chain to ensure the safety of consumers (Kshetri, 2019; Durrant et al., 2021; Jabbar et al., 2021). The onset of the COVID-19 pandemic has accelerated the application of blockchain technology in a multitude of areas. These applications are well-adept in addressing the challenges of future pandemics. Through these tactical approaches in the management during a pandemic, the multi-robot collaboration is designed in a heterogeneous and homogeneous fashion for specific non-contact tasks to be performed (Alsamhi and Lee, 2020). These include the monitoring of temperature, the delivery of goods and medical equipment both in an enclosed and open setting. This application can be extended to cover dual roles in the monitoring of food and crop supplies as well as the provision of them during a pandemic.

Blockchain technology further provides support for AI by facilitating integrated data management in a secure and fast manner, specifically when there is a data drive from multiple farm resources like underground sensors, weather stations, drones, irrigations systems, and related platforms. Supply chain management resources could be substantially supported by blockchain in agricultural context (Singh and Singh, 2020; Ordóñez et al., 2023). Promising results on Indian diary supply chain management were reported in a study where authors investigated the role of blockchain, distribution, and inventory management (Kumar and Kumar, 2023). Likewise, a Midwestern United States of America (USA) based company employed blockchain technology to enhance the production and supply chain of eggs, tracking their journey from farm to consumer tables (Bumblauskas et al., 2020). Nevertheless, the application of these technologies is somewhat limited in agriculture field. A review study published in 2020 revealed that only 20% of the published articles integrate blockchain with IoT and AI (Singh and Singh, 2020).

Besides, AI could be considered as a supplementary technology in weed identification, management of forestry and livestock, farm robotics, agricultural remote sensing (RS), wireless sensor networks for agriculture, mechanical pollination, and crop insurance for boosting agriculture production to meet the demands of the populations vulnerable to hunger. The advantage of agricultural RS is that it has been extensively researched in crop cultivation, monitoring, disease detection, and crucial areas of plant stress (DeChant et al., 2017; Fuentes et al., 2017; Liu et al., 2017; Lu et al., 2017; Ramcharan et al., 2017; Ghosal et al., 2018; Jin et al., 2018; Rançon et al., 2018; An et al., 2019; Barbedo, 2019; Brahimi et al., 2019; Cruz et al., 2019; Liang et al., 2019; Too et al., 2019; Ahmad et al., 2020; Esgario et al., 2020; Krishnaswamy Rangarajan and Purushothaman, 2020; Martos et al., 2021; Ahmad et al., 2022b).

## 5 Edge intelligence

In this section, we delve into the transformative realm of edge intelligence within the context of agriculture, exploring its applications, technological foundations, and the challenges it poses. The discussion unfolds with an insight into the emergence of edge intelligence as a dynamic AI application, strategically positioned at the network edge to meet the evolving demands of Beyond fifth Generation (B5G) networks. The primary focus lies on its pivotal role in enhancing agricultural practices, particularly in the optimization of food supply sustainability through efficient crop monitoring.

Emerging technologies such as edge intelligence have gained traction in the application of AI closer to the network edge in support of B5G needs. Edge intelligence is an alternative AI application that can be effectively employed in the monitoring of crops enabling a more efficient and sustainable food supply. Edge intelligence represents the convergence of AI and edge computing. Currently, a pivotal and widely employed methodology in this domain involves model compression, with a particular focus on techniques such as parameter pruning and quantization. The resources often used for edge intelligence include Central Processing Units (CPUs), Graphics Processing Units (GPUs), and Field Programmable Gate Arrays (FPGAs) (Liu et al., 2021). Edge intelligence facilitates the execution of AI analytics on intelligent edge devices closer to the source. Recently, pine wilt disease was detected using edge computing (Li et al., 2021). Likewise, applications like real-time weed mapping and immensely fast image processing have also been reported (Deng et al., 2020; de Camargo et al., 2021; Liu and Wang, 2021).

The arising concept of edge AI, involving the execution of AI models on edge computing devices, is instrumental in reducing the volume of data transmitted to the cloud (Gia et al., 2019). Notably, Shekhar et al. (2017) proposed a method utilizing a Raspberry Pi equipped with an AI model for automatic irrigation in agricultural fields. However, this approach lacks consideration for operations in elevated-temperature environments, such as the exceptionally high temperatures experienced during the summer in a greenhouse dedicated to tomato cultivation. The assessment of the automatic irrigation system revealed that the Jetson Nano, functioning as an edge node, exhibited viability for both image processing and irrigation judgment processing, showcasing resilience in environments with temperatures reaching up to 50°C (Kawai and Mineno, 2020). Consistent with these findings, previous studies have highlighted the applicability of Jetson Nano in plant stress detection and decision-making for subsequent spray operations (De Oliveira et al., 2022). Notably, recent research demonstrated an effective implementation of edge computing, employing a wireless sensor network to regulate micro-climatic parameters—such as temperature, soil moisture, light, pH, and salinity—within a greenhouse setting. The authors underscored that the integration of edge computing resulted in a substantial reduction in latency and diminished reliance on continuous cloud connectivity, thereby augmenting the overall responsiveness of the system (Rani et al., 2023). These outcomes collectively emphasize the versatility and efficacy of edge computing, particularly utilizing Jetson Nano, in diverse agricultural applications, ranging from irrigation management to plant stress detection and environmental parameter control within greenhouses. One of the prospectives use of edge intelligence could be for retrieving analyzed data statistics on mobile application from wireless sensor networks for extremely huge farms.

The use of edge intelligence in the context of Agriculture 5.0 is projected to take the form of data gathering (Alsamhi et al., 2021). On the other hand, Federated Learning (FL) enables decentralized collaborative learning through the development of localized models with the common use of model parameters within close proximity and the centralized unit in the improvement of accurate models (Alsamhi et al., 2021). In the context of our discussion, this will apply to the large area of crops across vast regions and countries. Nevertheless, drone edge intelligence presents its challenges particularly in security and a decentralized management which in turn results in limitations to its intended functions. Alternatively, blockchain technology allows for data sharing while maintaining its privacy and traceability (Durrant et al., 2021).

Despite the manifold advantages that edge computing brings to agriculture, its application is not without limitations. One noteworthy constraint lies in the constrained processing power of microcomputers when juxtaposed with robust cloud servers, potentially influencing the intricacy of locally executable algorithms. Furthermore, the scalability of edge computing systems may encounter challenges, particularly in the deployment and management of numerous edge devices across expansive agricultural landscapes. The integration of edge computing solutions with existing agricultural systems and machinery presents complexity, marked by potential compatibility issues when interfacing various sensors, machines, and edge devices. Additionally, the deployment and maintenance costs associated with edge computing infrastructure demand careful consideration, particularly in the context of small-scale or resource-constrained agricultural operations.

For a better comprehension of these challenges, consider a scenario where a wireless sensor network, leveraging edge computing in a vast agriculture field, confronts limitations in both processing power and storage capacity. This becomes particularly evident when grappling with extensive datasets generated by multiple sensors. The ramifications are tangible, affecting the real-time processing, and analytical capabilities of the deployed edge devices. Consequently, such limitations have the potential to impede the efficiency of decision-making processes, particularly in precision agriculture applications.

## 6 Quantifiable and measured benefits of AI

This section of the manuscript delves into the tangible and measurable advantages brought about by the integration of AI within the agricultural sector, with a specific focus on the economic potentials under the umbrella of Agriculture 5.0.

Beneficial outcomes remain at the core of each initiative in the progress of Agriculture 5.0. However, the COVID-19 pandemic has dampened progress in this area. As highlighted earlier (see Section 3), the European Green Deal might be the way out of such arduous circumstances. To quantify this viewpoint, the European Union has committed a third of the €1.8 trillion (approximately $2.1 trillion) investment from the NextGenerationEU Plan, and its seven-year budget to finance the European Green Deal (Commission, E, 2021a). This commitment will witness a reduction in net greenhouse gas emissions at a minimum threshold of 55% by 2030 with 1990 levels as the benchmark (Commission, E, 2021b). Whereas, by the year 2030, the projection of economic benefits from AI for Asia, including China, and North America is nearly 70% of the estimated $15.7 trillion (Arakpogun et al., 2021). In an analogous economic projection, the integration of AI technologies is anticipated to propel Brazil’s gross value added to $3,884 billion by the year 2035 (Kshetri, 2020).

How does this translate into the tangible benefits in the Agricultural sector? Consumers will experience healthy food that is affordable. This aligns with the objectives outlined in “A healthy food system for people and the planet” action plan (Commission, E, 2021c). The essence of the European Green Deal establishes a strong link between healthy people, societies, and the planet, enabling the European Union’s sustainable and inclusive growth strategy. The Common Agricultural Policy, a global standard in terms of safety, security of supply, nutrition, and quality is a pillar of the European agriculture and food system. Increasingly, this is being proposed as a standard for sustainability, having a positive effect on a sustainable food system. This will set the stage for balance, ensuring environmental-, health-, and social- benefits and economic profitability. For instance, analysis of data derived from crop production has the potential to enhance the annual global profit of significant agribusiness entities by an approximate sum of $20 billion (Bunge, 2014).

## 7 Addressing food safety and security through AI

A continuous and pathogen-free food supply, in other words food safety and security, is part of UNSDG. However, it remains a global concern and climate change is a notorious adversary to this goal. The WHO’s 2015 report indicated that on an annual basis, one in every 10 individuals falls ill due to the consumption of food tainted with microbial or chemical agents. This could lead to approximately 600 million instances of illnesses, causing around 420,000 fatalities and a collective loss of 33 million years of healthy life worldwide (WHO, 2022). Nevertheless, it has been documented that the African region bears the highest burden of foodborne diseases. To be more precise, the incidence of foodborne illnesses resulted in 1,200–1,300 Disability-Adjusted Life Years (DALYs), a public health metric for estimating the burden of disease, per 100,000 inhabitants in the year 2010, which is in stark contrast to the range of 35–711 observed in other regions (Pires et al., 2021). Soil is another natural resource that is important for food security, accounting for approximately 98.8% of our food supply. Nevertheless, climate change, loss of organic matter, greenhouse gasses emissions, intensification of agriculture, salinization, acidification, over application of fertilizers, and loss of biotic diversity are few of the most important threats to the food security (Kopittke et al., 2019; Ahmad et al., 2022a). Inadequate infrastructure, poverty and inequality, the impact of climate change, conflicts, and insufficient investment could be the primary factors contributing to the disparity between developing nations and developed nations in their efforts to ensure food security.

The use of AI could pave the way for ensuring food safety and security not only in developing countries but also in developed nations. Among various AI applications are early warning of outbreaks, risk predictions, monitoring and characterization of foodborne pathogens using computer vision, ML, and natural language processing (Qian et al., 2023). Nevertheless, substantial research and development endeavors persist within this domain. A majority of AI applications either exist in a state of partial development or are entirely nascent in their conception.

Furthermore, the application of AI among the solutions to food security involves the adoption of regenerative agriculture and permaculture (McLennon et al., 2021). McLennon et al. (2021) illustrated that specialized monoculture cropping systems, though meeting immediate food and fiber requirements, exhibit adverse impacts on natural resources, particularly affecting the sustainability of production agriculture. Focusing on regenerative agriculture, permaculture, and smart technology, the study advocated a holistic approach aimed at reducing dependence on external inputs, such as agrochemicals and machinery, to restore and maintain natural systems. Authors indicated that the proposed adoption of modern regenerative agriculture and integrated permaculture is anticipated to enhance soil health, biodiversity, land and resource conservation, agricultural sustainability, and global food security. The integration of digital agriculture and sustainable agricultural management, supported by contemporary agricultural technologies and data science (AI or ML), is deemed crucial for achieving these objectives.

Another aspect of supporting food safety and security could be the higher food production by increasing crop yields through AI technologies. The increase in crop yield through the application of AI has been widely applied. This application through the use of Earth Observation (EO) technology allows for large scale crop monitoring as frequently as once a day (Castillejo-González et al., 2009; Heupel et al., 2018; Potgieter et al., 2021). This application is ideal for the identification of the phenological stage of the crop (Potgieter et al., 2021). Of particular note is the use of single-date RS imagery that has been found to be efficient (Langley et al., 2001; Potgieter et al., 2017; Saini and Ghosh, 2018; Saini and Ghosh, 2021). Consequently, this could lead to the earlier identification of biotic and abiotic stresses in plants, which would help in decision-making. Crop monitoring and a timely identification of disease could contribute to saving on input costs, fertilizers applications, postharvest management; thereby increasing crop yield.

Additionally, multiple investigations have documented the utilization of multi-temporal RS techniques in discerning crop phenotypes, land cover, monitoring soil organic matter, predicting soil moisture, plant biomass modeling, and crop monitoring (McNairn et al., 2002; Upadhyay et al., 2008; Masjedi, 2020; Masjedi et al., 2020; Fathololoumi et al., 2021; Martos et al., 2021; Potgieter et al., 2021; Ma et al., 2023). These techniques facilitate precise prognostications to achieve predefined yield objectives by undertaking appropriate management approach subjected to analysis using AI algorithms, particularly deep learning methods such as CNN, as well as a feature fusion network. Such methods are commonly employed to extract meaningful information and patterns from complex and high-dimensional RS data.

In summation, the effectiveness of AI algorithms in the realms of precision agriculture, crop monitoring, pest and disease identification, input resources optimization, data analytics, and crop yield simulation has been substantiated through empirical evidence. Specifically, the integration of AI methodologies into areas such as supply chain optimization, food quality assessment, DSS, detection of specific food contaminants, sustainable land use planning, and simulations for food crises holds the potential to significantly elevate food safety and security levels in developed nations while concurrently providing substantial support to developing countries.

## 8 Challenges in the adoption of AI

Several factors influence the adoption of AI for agricultural operations in developing countries. Although the developed nations have not explored or reached the maximum potential of AI technologies, they certainly lead the industry. Several studies have discussed the technical challenges in the adoption of AI technologies in agricultural domains (Aly, 2020; Goralski and Tan, 2020; Zha, 2020; Bhat and Huang, 2021; Dwivedi et al., 2021; Smuha, 2021; Williamson et al., 2021; Ahmad and Núñez, 2022; Qazi et al., 2022; Whig, 2023). However, the complexities faced by developing nations in the assimilation of AI for agricultural purposes are multifaceted, deriving influence from socio-cultural, religious, ethical, economic, and cultural considerations. Therefore, considering the scope of present study, we highlighted the challenges faced in the adoption of AI technologies by dwelling on the previous studies, with their prospective solutions. These challenges concurrently encompass a subset of the constraints experienced by farmers in developed countries (Table 2).

- Financial challenges pose a significant hurdle in adoption of not only AI technology but also other farm technologies for developing nations. Implementation of AI technology, in particular, requires heavy upfront investment in software and hardware resources. Small scale farmers cannot afford these heavy costs without any subsidy program from local or international authorities. Likewise, the ongoing management and customization costs for local agricultural firms may involve additional costs. Besides, AI could be considered a quiet new technology for the farmers in developing countries, consequently, they might be hesitant to invest due to uncertainties about return on investment and potential risks. The possible solutions to these challenges include educating investors and farmers about the potential returns on investment from AI. On the other hand, government, and non-governmental organizations (NGOs) could also provide subsidies and microloans tailored to technology adoption. Similarly, establishing community centers where farmers could access AI tools and resources could save them from immense individual costs. In a similar manner, AI technology could be offered on rental or subscription basis to reduce the upfront costs.
- Infrastructure presents a huge challenge in the adoption of AI. Limited access to electricity hinders the execution of AI-driven devices. The lack of high-speed and stable internet connection prevents data transmission and cloud computing access. Similarly, the limited availability of hardware resources, i.e., computers, drones, servers, and sensors also impede the implementation of AI technologies. Besides, the absence of local support infrastructure could result in maintenance delays. Similarly, budget constraints for maintenance costs are another limiting factor. Nonetheless, initiatives involving international agencies, local governments, NGOs, and private sector entities to improve electricity and internet access, training and awareness could help tackle this challenge.
- Shortage of professionals poses a tremendous barrier to the adoption of AI in the agriculture sector. Agricultural communities often lack people with expertise in agricultural AI, which poses a barrier to developing, implementing, and maintaining AI systems. Likewise, deprivation of adequate training and capacity building programs along with non-AI-focused courses leave potential users without opportunities to equip themselves with essential knowledge. Language barriers and lack of local technical experts in AI makes it difficult to customize AI systems as per local needs. However, these challenges could be addressed in a number of ways including skill development and knowledge dissemination. Similarly, collaboration with renowned institutions and companies working in the domain of AI could help gain some training and support. Likewise, the translation of available literature on AI resources to the local language would also encourage people to further explore and learn about this technology.
- Absence of historical data, data scarcity and insufficient data availability poses a significant challenge in the adoption of AI technology for agriculture. Most of the AI systems need historical and real-time data about soil condition, crop growth, weather, and other relevant parameters for AI-driven processes. Existing data might not be available in the integrated or centralized form due to lack of cooperation among public and private sector. In addition, unstructured, inaccurate, outdated, and incomplete data could result in flawed insights. A possible remedy to encounter such challenges would be encouraging collaboration between farmers, local agricultural ministry, and research institutions to share and record data according to the established data standards and protocols. The small volume of incomplete data could be improved by techniques like data synthesis and imputation to avoid flawed AI-decisions.
- Customization is another challenge in the adoption of AI technology, revolving around the need to personalize AI systems according to the languages, agricultural practices, and contexts of specific regions. Crops, agronomic practices, crops diseases, and pests vary a great deal across regions, which means a universal AI model fit for all purposes cannot be devised. Besides, traditional agricultural practices could be vital for the success of a particular crop, which necessitates the integration of indigenous knowledge into AI solutions. Local policies and regulations might also require customization of AI systems. A holistic approach might be needed to address customization challenges. Devising AI solutions by the involvement of local farmers, extension workers, agronomists, agricultural researchers, economists, policy makers, and AI experts could ensure effective solutions effectively addressing farmers’ needs and contributing to improved productivity and sustainability in farming practices. Additionally, designing user-centered AI interfaces with region specific information could enhance the adoption rates and relevance of AI models.
- Absence of proper regulatory framework is a significant challenge both for developed and developing countries. However, developed countries are swiftly taking actions in this regard. Developing nations face severe challenges related to policies, standards, and laws that may govern the deployment and use of AI technologies. Lack of specific regulations creates uncertainties for stakeholders regarding liabilities, obligations, and ethical considerations. Similarly, the continuously evolving and complex nature of AI technologies causes regulatory gaps and challenges in ensuring compliance for regulatory bodies. Besides, local data storage and processing of sensitive agricultural data could lead to concerns about data sharing, privacy, ownership, and infringement of farmer’s rights. AI applications raise several ethical questions regarding bias in transparency of algorithms, decision-making, and potential impacts on labor market. Apart from these challenges, compliance costs could be prohibitive for start-ups and small-scale farmers. Lack of expertise of the governing bodies is also a tremendous problem for devising regulatory framework for AI technology adoption. The potential solutions to address these challenges could involve a capable governing body establishment; creation of comprehensive regulations considering industry, academia, and civil society; the encouragement of international cooperation and harmonization; and developing ethical guidelines for the responsible use of AI.
- Cultural norms and attitudes could also pose skepticism toward AI adoption. Introduction of AI technology could threaten the generational long beliefs and farming practices in various developing regions. The fear of negative impacts or job displacement could also hinder the adoption of AI technology. Most of the time, farmers perceive technology as distant from their immediate concerns due to lack of awareness and low literacy levels. In order to resolve these challenges, solutions respecting local traditions and values could contribute toward trusting the new technology. Likewise, communicating AI benefits in the local language and narrating near to close examples or use cases could also contribute to AI technology acceptance. Encouraging participation of community leaders or influential people for technology comprehension with practical demonstrations could also result in greater acceptance and adoption.
- Access to market is another substantial challenge in the adoption of AI technology. Various developing regions encounter problems of lack of infrastructure for transportation, storage, and distribution facilities. Lack of market knowledge hinders farmers from making informed decisions on cultivation of specific crops and use of appropriate technology for higher revenue. Rural areas often face digital divide due to which it becomes impossible to access e-commerce platforms, online marketing, and financial services. Dearth of desired skills for marketing and networking could also keep some farmers from technology adoption. Nonetheless, strategic approaches could be adopted to address these challenges. Information platforms with real-time market prices and demand can be created for facilitating decision-making. Mobile applications development in local language for facilitating access to local and cross-border buyers could also be a possible solution. Furthermore, developing policy support by regulatory bodies could enhance trust, security, and access for buyers and sellers alike. Additionally, microlearning or small training sessions with practical examples could elevate the literacy rate and market negotiation skills among small-scale farmers.
- Lastly, lack of interdisciplinary collaboration could be another significant challenge in AI adoption for agriculture. Generally, AI implementation for agriculture requires cooperation among experts in data science, economics, agronomy, engineering, policy making, social science, and AI, which often lacks in developing countries. Lack of cross-training could lead to difficulties in identifying potential synergies. In addition, people from diverse backgrounds could have different priorities, goals, values, and cultural norms that could cause challenges in aligning respective perspectives. Bureaucratic organizational structures, time, and resource constraints, and linguistic barrier also pose sever hindrance in AI adoption. Considering the potential solutions, organization of interdisciplinary seminars with basic knowledge sharing of various fields could foster a collaborative environment. In this regard, incentivizing interdisciplinary cooperation could also motivate experts to work together. Defined project objectives and clear communication channels could also overcome the collaboration challenges.

## 9 Agriculture 5.0─the way forward

**Figure 7:**
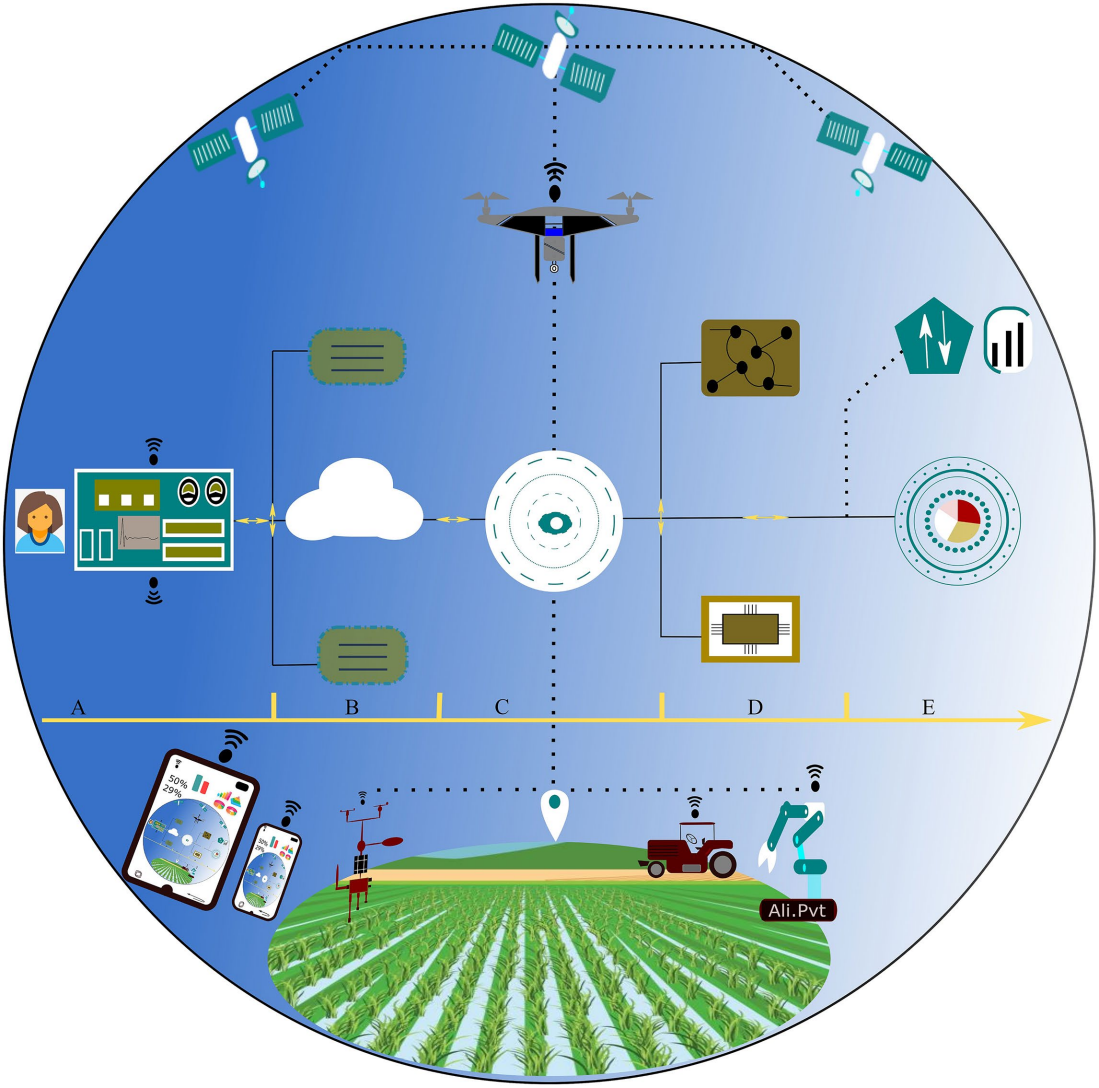
Conceptual illustration of AI-driven Agricultural Technology Strategy. The sequential flow, from **(A)** user control center to **(B)** servers, cloud, and associated storage devices, followed by **(C)** deployment of entities such as satellites, remotely piloted aircraft (RPAs), autonomous robots, meteorological stations, etc., for data acquisition. Subsequently, **(D)** involves data processing utilizing machine learning and associated platforms, ultimately yielding **(E)** infographics and pertinent information. The directional progression is from left to right.

The fifth industrial revolution holds immense potential for the agriculture sector. The applications of Agriculture 5.0 are wide and have been researched and deployed in fields, farmlands, vineyards across continents. Especially, AI like emerging technologies is increasing the efficiency of current and traditional farming systems. Considering the scientific reports discussed earlier in this study, it would be safe to argue the manifold benefit of AI. AI could bridge the hunger gap by efficient use of resources and increasing crop yield. Developing regions, in particular, could contribute a great deal to future food demand. Since Agriculture 5.0 is about resilient crop cultivation programs where an integrated approach for farm technology is suggested, therefore employment of AI is of prime importance for execution, management, processing, and coordination tasks. In this integrated approach, AI assumes a pivotal role by enabling task allocation and conducting data analytics, culminating in the production of outcomes presented through a mobile application or a user-friendly interface (Figure 7).

Given the unique challenges faced by developing countries, initiating small-scale projects, such as community-based agricultural programs or local cooperative efforts, could significantly contribute to advancing their journey toward building sustainable and resilient agricultural systems. In this regard, the feasible way would be to employ the technologies that have been developed and used in advanced economies through collaborative partnerships or as pilot programs. The optimization of these technologies could enhance and uplift current food production in the developing world for domestic consumption and for trade in the global food market. Leveraging Agriculture 5.0 with the capabilities of AI is certain to bridge the deficiency of food supplies in regions where malnourishment remains persistent.

**Table 2:** Summary of significant challenges in the adoption of Artificial Intelligence (AI) in developing and under-developed regions.

Challenge	Description	Recommendations
Financial challenges	Heavy upfront investment in software, hardware, and sensors. No subsidy program from local or international authorities. Additional management and customization costs. Lack of funding by investors due to uncertainties about return on investment.	Educating investors about the potential returns on investment from AI. Government and non-governmental organizations (NGOs) based initiatives for technology adoption. Collective acquisition of technology tools through community centers. Rental or subscription services to reduce the upfront costs.
Infrastructure	Inadequate access to electricity along with the lack of high-speed and stable internet. Limited availability of AI hardware resources, e.g., servers, sensors, etc. The absence of local support due to budget constraints.	Initiatives involving international agencies, local governments, NGOs, and private sector entities to improve electricity and internet access. Training and awareness creation.
Field experts	Lack people with expertise in agricultural AI, which poses a barrier to developing, implementing, and maintaining AI systems. Deprivation of adequate training. Language barriers to customize AI systems as per local needs.	Skill development and knowledge dissemination. Training support through collaboration with renowned institutes and companies. Translation of available literature on AI resources to the local language.
Data availability	Absence of historical data about soil condition, crop growth, weather, and other relevant parameters. Data scarcity and insufficient centralized data. Incomplete data could result in flawed insights.	Encouraging data recording according to established standards and protocols. Incomplete data should be improved by techniques like data synthesis and imputation to avoid flawed AI-decisions.
Customization	Inapplicability of AI model due to diversity in crops, agronomic practices, crops diseases, and pests across regions. Lack of integration of indigenous knowledge about farming. Incompliance of AI solutions to local policies and regulations.	Devising AI solutions by the involvement of local farmers, extension workers, agronomists, agricultural researchers, economists, policy makers, and AI experts. Designing user-centered AI interfaces with region specific information.
Regulatory framework	Lack of specific regulations creates uncertainties for stakeholders. Regulatory gaps and challenges due to the continuously evolving and complex nature of AI technologies. Concerns about data sharing, privacy, ownership, and copyrights infringement. Ethical apprehensions regarding bias in transparency of algorithms, decision-making and potential impacts on labor market. Lack of expertise of the governing bodies in devising regulatory framework for AI technology adoption.	Establishment of a capable governing body. Creation of comprehensive regulations keeping in mind industry, academia, and civil society. Encouraging international cooperation and harmonization Formulation of ethical guidelines for responsible use of AI.
Cultural norms and attitudes	Perceived threat to the generation long beliefs and farming practices. Fear of negative impacts or job displacement. Perception of technology as distant from the immediate concerns.	Customized AI solutions respecting local traditions and values. Communication of AI benefits in the local language and narrating near to close examples or use cases. Encouraging participation of community leaders or influential people for technology comprehension.
Access to market	Lack of infrastructure for transportation, storage, and distribution facilities. Dearth of market knowledge to make informed decision on cultivation of specific crops and use of appropriate technology for higher revenue. Severe digital divide in rural areas hindering access to e-commerce platforms, online marketing, and financial services. Lack of networking and marketing skills among small-scale farmers.	Creation of information platforms with real-time market prices and demand for facilitating decision-making. Development of mobile applications in local language for facilitating access to local and cross-border buyers. Devising policy support to enhance trust, security, and access for buyers and sellers alike. Organizing microlearning or small training sessions with practical examples to elevate the literacy rate and market negotiation skills among small-scale farmers.
Interdisciplinary collaboration	Absence of cooperation among experts in data science, economics, agronomy, engineering, policy, social science, and AI. Lack of cross-training leads to difficulties in identifying potential synergies. Diverged perspectives due to different priorities, goals, values, and cultural norms. Bureaucratic organizational structures, time, and resources constraints.	Organization of interdisciplinary seminars to foster a collaborative environment. Incentivizing interdisciplinary cooperation. Setting up well-defined project objectives. Establishing clear communication channels to overcome the collaboration challenges.

In the context of Sub-Saharan African region, staple crops reported for this region include plantains, cassava, corn, maize, millet, and sugarcane (Fatema et al., 2019). A comprehensive depiction of the geographical distribution of crop types in relation to regions that experience chronic hunger had been reported previously (Programme, 2021). Notably, the two major crops, i.e., maize and cassava, are prevalent in the countries most afflicted by hunger in Sub-Saharan Africa. This suggests that a targeted approach utilizing a limited number of technologies could potentially yield the desired outcomes of ensuring ample food supplies for the malnourished population. In light of identifying regions most susceptible to hunger, the imperative consideration for these vulnerable areas lies in the implementation of extensive Agriculture 5.0, harnessing artificial intelligence technologies. These staple crops, which were a subset of those studied by several researchers (DeChant et al., 2017; Liu et al., 2017; Lu et al., 2017; Ramcharan et al., 2017; Ghosal et al., 2018; Rançon et al., 2018; Barbedo, 2019; Brahimi et al., 2019; Too et al., 2019; Esgario et al., 2020; Krishnaswamy Rangarajan and Purushothaman, 2020) have paved the way for the implementation of RS on a large scale within this region taking into account various factors, including weather conditions, climate variations, specific crop types, and production goals, to optimize the selection of appropriate systems and technologies.

In line with the insights from Sections 4–6, the proposed AI technologies have undergone comprehensive research, establishing a promising use case for Sub-Saharan Africa. These technologies, along with their corresponding execution models, can be further developed and disseminated globally following successful implementation in these regions. Given the extensive agricultural landmass, this approach offers the potential to amass substantial data, enhancing efforts to refine and expand AI applications both within Sub-Saharan Africa and beyond.

## 10 Conclusion

The objective of this study was to explore the in-depth potential of AI in the agriculture sector for developing and under-developed countries. Similarly, it aimed to emphasize the proven efficiency and spin-off applications of AI in the advancement of agriculture. Considering the soaring world population, increasing food demand, and climate change, AI could be identified as a plausible technology in this 5th industrial revolution in bringing us closer to achieving zero hunger by 2030—Goal 2 of the UNSDG. At present, AI is being utilized in various spheres of agriculture, including but not limited to crop surveillance, irrigation management, disease identification, fertilization practices, task automation, image manipulation, data processing, yield forecasting, supply chain optimization, implementation of DSS, weed control, and enhancement of resource utilization, among a multitude of other applications. In a similar manner, AI supports food safety and security by ensuring higher crop yields.

Furthermore, various challenges in the adoption of AI for developing nations have been identified with their subsequent remedies. These include constraints like financial, infrastructure, expertise, data availability, customization, regulatory framework, cultural norms and attitudes, access to market, and interdisciplinary collaboration. To effectively address the challenges encountered by developing countries in the integration of AI within the agricultural sector, a comprehensive approach involves the strategic formulation of policies, targeted investment initiatives, the enhancement of existing infrastructure, fostering skill development and training programs, promoting widespread awareness, and tailoring AI solutions to cater to user needs. Moreover, the establishment of ethical guidelines to govern AI implementation, alongside fostering international collaboration and knowledge exchange, emerges as crucial component to combat these challenges. The identification of challenges and opportunities in the implementation of AI could ignite further research and actions in these regions.

## Author contributions

AA: Conceptualization, Data curation, Investigation, Methodology, Validation, Visualization, Writing – original draft, Writing – review & editing. ALi: Methodology, Writing – original draft. FV: Investigation, Writing – original draft. AK: Investigation, Writing – original draft. AC: Investigation, Writing – original draft. GB: Investigation, Writing – original draft. SA: Investigation, Writing – original draft. PC: Investigation, Writing – original draft. PR: Investigation, Writing – original draft. ALy: Investigation, Writing – original draft. MG: Investigation, Writing – original draft. CX: Investigation, Writing – original draft. RLB: Investigation, Writing – original draft. ID: Investigation, Writing – original draft. IE: Investigation, Writing – original draft. GD'U: Investigation, Writing – original draft. LG: Writing – review & editing, Supervision, Investigation. VM: Conceptualization, Funding acquisition, Project administration, Resources, Validation, Writing – original draft.
